# It Ain’t What You Do (But the Way That You Do It): Will Safety II Transform the Way We Do Patient Safety? Comment on "False Dawns and New Horizons in Patient Safety Research and Practice"

**DOI:** 10.15171/ijhpm.2018.14

**Published:** 2018-02-18

**Authors:** Rebecca Lawton

**Affiliations:** ^1^School of Psychology, University of Leeds, Leeds, UK.; ^2^Bradford Institute for Health Research, Bradford Teaching Hospitals Foundation Trust, Bradford, UK.

**Keywords:** Patient Safety, Systems Thinking, Safety I, Safety II, Resilience

## Abstract

Mannion and Braithwaite outline a new paradigm for studying and improving patient safety – Safety II. In this response, I argue that Safety I should not be dismissed simply because the safety management strategies that are developed and enacted in the name of Safety I are not always true to the original philosophy of ‘systems thinking.’

## Introduction

In the late 1980s, 1 in 1000 shunters (the railway staff who couple and uncouple trains) were killed working on the railway in Britain each year. In 1991 I embarked on a PhD exploring why shunters broke the rules that were there to keep them safe. I published the work in an article entitled ‘Not working to rule: Understanding procedural violations at work.’^1^ While not my finest work, I am now, with the publication of Mannion and Braithwaite’s article,^2^ reinvigorated by the idea that I may have been one of the first people to really try to understand ‘work as done.’^3^ In fact, in the abstract of the paper I write: *Generally, violations were perceived to be the result of a well-intentioned desire to get the job done.* Shunters were not taking risks with their own lives because they were macho, thrill-seeking men, but because two-way radios were not working, sidings had curves in them (obscuring their view of the driver), shunting poles were sometimes difficult to use to couple the trains, requiring them to stand between carriages instead. The approach I took to studying shunters in situ, training to be one myself and spending many hours in shunting cabins, resonates absolutely with the emerging methods (ethnography^4^) and premise of Safety-II, although the language is quite different. I rather embarrassingly refer to violations, the nomenclature of the time, but I much prefer the less punitive and more subtle ‘work as done.’ My supervisor and mentor in this work was Professor James Reason, of Swiss-cheese and organisational-accident model fame.^5^ He, along with Lucian Leape in the United States,^6^ was one of the founders of the systems approach to safety, as pointed out in the Mannion and Braithwaite article. James Reason was an inspirational supervisor, so when he recommended that I immerse myself in shunting, don steel toe capped boots and a high visibility vest each morning, I did not question his motives. He recognised that to understand why people behave the way they do one needs to understand the person in their social and organisational context; in other words within the wider system. So this is what I did. I tell this story because I am not convinced that it is the Safety-I theories or models that are to blame for our lack of progress, but rather the operationalisation of these within healthcare systems. So here I ask were the dawns false or have we just ignored their eerie light? Let’s take one example, learning from past harm, to help elucidate this argument.

Many healthcare systems place ‘incident reporting’ at the heart of their safety management armoury, the primary purpose being to learn from these past harms. These systems suffer from low reporting rates^7^ and limited learning that appears to be related, at least in part, to poor feedback mechanisms.^8^ Even more problematic, however, is that organisations (and wider systems) rarely act on the incident data to do those things that, according to systems thinking, are likely to make a difference to safety in the future – redesigning tasks and work-processes, promoting teamwork and interdisciplinary communication, redesigning equipment or workplaces, and developing leaders that support staff wellbeing.^9^ The problem is one of both poor quality incident investigations and a failure to act on those contributory factors identified. Indeed, some responses to incidents, such as writing new procedures, disciplining and retraining staff, serve only to add to the system problem and often show little resemblance to the contributory factors identified in the incident investigation. We are now moving into an era where we recognise that measuring and monitoring safety is about much more than just past harm^10^ and, of course, it is naïve to think that incident reporting systems are the panacea of patient safety. However this shift in thinking away from a focus on past harm is not a function of Safety-II. In fact, the idea of frontline staff requiring ‘error wisdom’ and acting on the here and now was promoted within Safety I by Reason^11^ amongst others.

Patient safety has also suffered, at least to some extent, from the application of a clinical lens. Patient safety incidents, like diseases and illnesses have been categorised and treated. So many of the interventions we have developed tackle specific negative outcomes (eg, falls prevention, pressure ulcer prevention, sepsis prevention, infection prevention) yet so many of the factors contributing to incidents (inter-professional rivalry, work environment, equipment that is poorly designed, poor staffing levels and high workload) are not unique to a particular outcome, they are common across all. Local safety/improvement projects may demonstrate improvements eg, a reduction in falls on a hospital ward, by encouraging staff to more reliably enact a process. However, the consequences of these interventions might not always be as anticipated – other patient safety outcomes might be ignored. For example, patients might return home with reduced mobility because their movement in hospital has been so restricted that they are now deconditioned. This, in turn increases their likelihood of falling within the home. In other words, we have simply shifted the problem of falls to elsewhere in the system.

What we have failed to understand is that very many of the system factors that underpin safety are not amenable to local change and some are not amenable to organisational change, instead requiring intervention from those funding professional training or designing and manufacturing medical devices or setting targets. Others have written eloquently on this subject,^12^ but for me the simple metaphor that we have focused our attention on swatting mosquitoes, rather than on draining swamps, is sufficient. To blame Safety-I and systems theory for the ‘quick fix’ approach to dealing with safety problems is difficult to justify.

Systems thinking may have, as Mannion and Braithwaite suggest, been embraced by those who seek simple cause-effect relationships and espouse the use of root cause analysis, but the originators were much more sophisticated in their thinking. There is nothing simplistic and linear in the following explanation of systems thinking in ‘An Organisation with a memory’ (2000).^13^

*The system approach … recognises that many of the problems facing organisations are complex, ill-defined and result from the interaction of a number of factors.… Errors are seen as being shaped and provoked by ‘upstream’ systemic factors, which include the organisation’s strategy, its culture and the approach of management towards risk and uncertainty…. The system approach recognises the importance of resilience within organisations and also recognises the process of learning as enhancing such resilience*.

The term resilience is now associated with Safety-II and is used in the title of a number of Jeffrey Braithwaite’s series of books, but here it is clear that the term is being used very much within the Safety I paradigm. The inquiries into major disasters of the 1980s and 1990s also demonstrate this sophistication in thinking. For example, Lord Cullen^14^ who led the inquiries into both Piper Alpha and Ladbroke Grove disasters writes:

“*I examined the significance of whatever had a tenable connection with the chain of events which led up to the catastrophe, and I also took account of other factors which played no part in bringing about the result, but which were in themselves cause for concern.*”

What Lord Cullen recognises in this comment is that a negative outcome is actually an opportunity to gain a better understanding of the way the complex socio-technical system works to produce the outcomes it does. However, what differs from a Safety II perspective is that the adaptations that are made to work around/ cope with system failures are construed more positively as resilient behaviours rather than errors.^15^ By so doing, it may be possible to reduce the shame and blame response that still exists in healthcare.^16,17^

So, while I agree with Mannion and Braithwaite that ‘top-down, standardised policy, regulations and linear-style interventions’ are limited in their effectiveness, I do not agree that systems thinking, as initially conceived at least, supports this way of doing things. Indeed my experience of working within this paradigm has led to interventions that promote the patient voice^18^ or empower the staff to deliver change.^19^ In other words, I think it is the way we have ‘done’ Safety I and continue to do so that is the problem, rather than that the founding principles of Systems thinking are fundamentally flawed. Perhaps, by turning safety on its head and asking different questions about how we promote excellence, how we learn from what goes well, how we understand work as imagined and work as done, we will develop different ways of ‘doing’ safety. Intuitively it is hard to disagree with this sentiment and a shift towards this more positive language and message of Safety II seems to be of the time. Healthcare staff need to hear the message that excellence is possible, that they are doing a good job despite their working context, and that we recognise their resilience. This is a message that is too little heard as we try repeatedly to swat mosquitoes that just won’t go away.

However, we do not yet know what types of solutions and interventions Safety II might produce and indeed some proponents (Hollnagel, personal communication) believe that organisational resilience is an emergent property that we should understand but not expect to be able to engineer. There is, of course, the possibility that, with limited resources and a ‘command’ culture, Safety II will serve only to identify those ‘excellent’ things that others must aspire to and new rules and protocols spring up in an attempt to produce more ‘excellence.’ Another possible future is one where we identify and praise resilience amongst staff, teams and organisations and we fail to improve, simplify and transform those systems that require staff to repeatedly adapt and flex and find workarounds. Neither of these two possible futures are desirable.

If, on the other hand, Safety II is the beginning of a different ‘way of doing things’ where staff feel they can lead change and improve from the bottom-up the imperfect system in which they work, then I am with Mannion and Braithwaite all the way.

## Ethical issues

Not applicable.

## Competing interests

Author declares that she has no competing interests.

## Author’s contribution

RL is the single author of the paper.

## Disclaimer

This research was supported by the National Institute for Health Research (NIHR) Yorkshire and Patient Safety Translational Research Centre (NIHR YH PSTRC). The views expressed in this article are those of the author and not necessarily those of the NHS, the NIHR, or the Department of Health and Social Care.

## References

[1] Lawton R (1998). Not working to rule: Understanding procedural violations at work. Saf Sci.

[2] Mannion R, Braithwaite J (2017). False dawns and new horizons in patient safety research and practice. Int J Health Policy Manag.

[3] Braithwaite J, Wears RL, Hollnagel E. Resilient Health Care, Volume 3: Reconciling Work-as-Imagined and Work-as-Done. CRC Press; 2016.

[4] Anderson JE, Ross AJ, Back J (2016). Implementing resilience engineering for healthcare quality improvement using the CARE model: a feasibility study protocol. Pilot Feasibility Stud.

[5] Reason J (2000). Human error: models and management. BMJ.

[6] Leape LL, Bates DW, Cullen DJ (1995). Systems analysis of adverse drug events. ADE Prevention Study Group JAMA.

[7] Sari AB, Sheldon TA, Cracknell A, Turnbull A (2007). Sensitivity of routine system for reporting patient safety incidents in an NHS hospital: retrospective patient case note review. BMJ.

[8] Benn J, Koutantji M, Wallace L (2009). Feedback from incident reporting: information and action to improve patient safety. Qual Saf Health Care.

[9] Lawton R, McEachan RR, Giles SJ, Sirriyeh R, Watt IS, Wright J (2012). Development of an evidence-based framework of factors contributing to patient safety incidents in hospital settings: a systematic review. BMJ Qual Saf.

[10] Vincent C, Burnett S, Carthey J (2014). Safety measurement and monitoring in healthcare: a framework to guide clinical teams and healthcare organisations in maintaining safety. BMJ Qual Saf.

[11] Reason J (2004). Beyond the organisational accident: the need for “error wisdom” on the frontline. Qual Saf Health Care.

[12] Dixon-Woods M, Pronovost PJ (2016). Patient safety and the problem of many hands. BMJ Qual Saf.

[13] Donaldson LJ, Appleby L, Boyce J. An organisation with a memory: report of an expert group on learning from adverse events in the NHS. Norwich, United Kingdom: Stationery Office; 2000.

[14] Cullen WD. The Ladbroke Grove rail enquiry: part 1 report. London: HMSO; 2001.

[15] Sujan M, Huang H, Braithwaite J. Why do healthcare organisations struggle to learn from experience? A safety-II perspective. Proceedings of Healthcare Systems Ergonomics and Patient Safety Conference (HEPS). Toulouse, France; 2016.

[16] Cooper J, Edwards A, Williams H (2017). Nature of Blame in Patient Safety Incident Reports: Mixed Methods Analysis of a National Database. Ann Fam Med.

[17] Cohen D (2017). Back to blame: the Bawa-Garba case and the patient safety agenda. BMJ.

[18] Lawton R, O’Hara JK, Sheard L (2017). Can patient involvement improve patient safety? A cluster randomised control trial of the Patient Reporting and Action for a Safe Environment (PRASE) intervention. BMJ Qual Saf.

[19] Taylor N, Lawton R, Slater B, Foy R (2013). The demonstration of a theory-based approach to the design of localized patient safety interventions. Implement Sci.

